# Immunohistochemical Characterization of M1, M2, and M4 Macrophages in Leprosy Skin Lesions

**DOI:** 10.3390/pathogens12101225

**Published:** 2023-10-09

**Authors:** Tatiane Costa Quaresma, Lívia de Aguiar Valentim, Jorge Rodrigues de Sousa, Tinara Leila de Souza Aarão, Hellen Thais Fuzii, Maria Irma Seixas Duarte, Juarez de Souza, Juarez Antônio Simões Quaresma

**Affiliations:** 1 Health Department, Center for Biological and Health Sciences, State University of Para-CCBS, UEPA, Belem 66087-662, Brazil; 2 School of Medicine, Federal University of Para-UFPA, Altamira 68440-000, Brazil; 3 Health Department, Tropical Medicine Center, Federal University of Para-NMT-UFPA, Belem 66055-240, Brazil; 4 School of Medicine, Sao Paulo University, Sao Paulo 01246-903, Brazil

**Keywords:** leprosy, macrophages, immunohistochemistry, immunology

## Abstract

*Mycobacterium leprae* is the etiological agent of leprosy. Macrophages (Mφs) are key players involved in the pathogenesis of leprosy. In this study, immunohistochemical analysis was performed to examine the phenotype of Mφ subpopulations, namely M1, M2, and M4, in the skin lesions of patients diagnosed with leprosy. Based on the database of treatment-naïve patients treated between 2015 and 2019 at the Department of Dermatology of the University of the State of Pará, Belém, routine clinical screening samples were identified. The monolabeling protocol was used for M1 macrophages (iNOS, IL-6, TNF-α) and M2 macrophages (IL-10, IL-13, CD163, Arginase 1, TGF-β, FGFb), and the double-labeling protocol was used for M4 macrophages (IL-6, MMP7, MRP8, TNF-α e CD68). To confirm the M4 macrophage lineage, double labeling of the monoclonal antibodies CD68 and MRP8 was also performed. Our results demonstrated a statistically significant difference for the M1 phenotype among the Virchowian (VV) (4.5 ± 1.3, *p* < 0.0001), Borderline (1.6 ± 0.4, *p* < 0.0001), and tuberculoid (TT) (12.5 ± 1.8, *p* < 0.0001) clinical forms of leprosy. Additionally, the M2 phenotype showed a statistically significant difference among the VV (12.5 ± 2.3, *p* < 0.0001), Borderline (1.3 ± 0.2, *p* < 0.0001), and TT (3.2 ± 0.7, *p* < 0.0001) forms. For the M4 phenotype, a statistically significant difference was observed in the VV (9.8 ± 1.7, *p* < 0.0001), Borderline (1.2 ± 0.2, *p* < 0.0001), and TT (2.6 ± 0.7, *p* < 0.0001) forms. A significant correlation was observed between the VV M1 and M4 (r = 0.8712; *p* = 0.0000) and between the VV M2 × TT M1 (r = 0.834; *p* = 0.0002) phenotypes. The M1 Mφs constituted the predominant Mφ subpopulation in the TT and Borderline forms of leprosy, whereas the M2 Mφs showed increased immunoexpression and M4 was the predominant Mφ phenotype in VV leprosy. These results confirm the relationship of the Mφ profile with chronic pathological processes of the inflammatory response in leprosy.

## 1. Introduction

Leprosy is an infectious disease with low morbidity owing to the population’s resistance to *Mycobacterium leprae* [1]. It has been demonstrated that during an inflammatory response, bone marrow-derived monocytes differentiate into macrophages (Mφs) to regulate innate and adaptive immunity, as well as maintain homeostasis in response to the development of inflammatory episodes during the course of the disease [2]. However, the immunological functions of Mφs are highly dependent on specific signals from antigen-specific immune cells and the microenvironment in which they reside [3].

Mφs are capable of undergoing phenotypic modification and expressing receptors and co-stimulatory molecules such as cytokines that induce the development of appropriate immunological responses [4]. Studies on Mφ polarization have demonstrated the relationship between M1 Mφs (MφM1) and MφM2 in leprosy [5,6,7].

Classical activation of Mφs occurs via IFN-γ stimulation, which generates MφM1 with high pathogen-killing potential and upregulates the secretion of pro-inflammatory cytokines such as IL-6, IL-12, inducible nitric oxide synthase (iNOS), and TNF-α. The expressed molecules or factors are Janus kinase 1 (JAK1), JAK2, signal transducer and transcription activator 1 (STAT1), and STAT2 [4]. In the absence of IL-12, the phenotypic profile of Mφ deviates from that of the MφM2 [4].

MφM2 play a key role in the resolution of inflammation, promoting the removal of debris and enabling an increase in the contractility of the smooth muscle, thereby contributing to the expulsion of pathogens [8]. Activation of MφM2 occurs when they are exposed to a microenvironment with IL-4 and IL-13 stimulation. MφM2 secrete IL-10, TGF-β, arginase-1 [4], and prostaglandin E2 [9], and express JAK1, JAK2, JAK3, and STAT6 [4].

MφM1 and MφM2 mainly differ with respect to their receptors, effector functions, and cytokine production. Upon induction by lipopolysaccharides (LPSs) or IFN-γ, activated MφM1 of the Th1 lineage express high levels of iNOS, which metabolizes arginine to nitric oxide and citrulline. In contrast, MφM2 of the Th2 lineage are characterized by their expression of arginase, which hydrolyzes arginine to ornithine and polyamines. Although M1/M2 polarization leads to opposing outcomes of inflammatory reactions, the balance between cytotoxicity (MφM1) and immunosuppression (MφM2) is vital for the homeostasis of the immune system [4]. A growing body of evidence suggests that a new Mφ subpopulation, known as M4, is associated with the development of pro-inflammatory responses, oxidative stress, and tissue repair in the polar forms of leprosy [4,10].

The advancement of knowledge on immunology in recent years has contributed to a deeper understanding of the mechanisms involved in the pathogenesis of leprosy. Due to the fact that macrophages are the main cells involved in the immune response to M. leprae, and due to the few comparative studies published in the scientific literature on the role of M1, M2, and M4 macrophage subpopulations in the pathogenesis of cutaneous lesions in the different clinical forms of leprosy, the objective of this work was to comparatively evaluate the presence and possible role of these cells using the immunohistochemistry technique.

## 2. Materials and Methods

### 2.1. Patients and Skin Lesion Samples

This is a cross-sectional study, in which we analyzed 42 samples of skin lesions obtained from patients treated at the Dermatology Service of the State University of Pará between 2015 and 2019, available on file, and therefore a sample of convenience. All patients were treatment-naïve and diagnosed with borderline (*n* = 14), TT (*n* = 14), or VV (*n* = 14) leprosy, according to Ridley and Jopling’s classification. Samples from underage patients, co-infected with HIV, who had presented reactions or who underwent a biopsy of the lesion after starting multidrug therapy, according to information obtained from the medical records, were excluded. Samples from patients diagnosed with borderline leprosy were also excluded, due to the immunological instability characteristic of this clinical form, which would compromise the analysis of the data obtained. Statistical analysis was performed using the BioStat 5.0 program. In the univariate analysis, frequencies and measures of central tendency and dispersion were obtained, together with ANOVA. A limiting significance level of 5% (*p* = 0.05) was adopted for all tests.

### 2.2. Immunostaining for Mφ Characterization and Statistical Analysis

The monolabeling protocol was used for M1 macrophages and M2 macrophages and the double-labeling protocol for M4 macrophages. M1 macrophages were characterized by immunostaining for monoclonal antibodies to iNOS, IL-6, and TNF-α. M2 macrophages were characterized by immunostaining for monoclonal antibodies to IL-10, IL-13, CD163, Arginase 1, TGF-β, and FGFb. M4 macrophages were characterized by immunostaining for monoclonal antibodies to IL-6, Metalloproteinase 7, MRP8, TNF-α, and CD68. To confirm the M4 macrophage lineage, double labeling of the monoclonal antibodies CD68 and MRP8 was also performed, following the protocol of Quaresma et al. [11]. We performed an immunostaining technique assay for detection, based on complex formation with EnVision horseradish peroxidase (HRP) polymer (Agilent, Santa Clara, CA, USA), after which the same sections were incubated with streptavidin–alkaline phosphatase complex (Invitrogen Corporation, Camarillo, CA, USA) 1:20 dilution for 1 h at room temperature, after which the slides were washed 3 times with 0.1 M TRIS solution. The HistoMark Red Phosphatase Kit (KPL, Gaithersburg, MD, USA) was applied to the slides for 30 min at room temperature for the detection of the antibody staining. By examining the lesion area using a 400× zoom in microscope Zeiss Axio Imager Z1lens, immunomarkers were quantitatively analyzed by selecting five random fields. Each field was subdivided into regions of 0.0625 mm^2^ within an area of 10 × 10 mm^2^ delimited by the microscope lens. Only samples showing cells immunostained for at least two markers were considered positive. For quantitative analysis of immunological markers, the average number of cells stained for double markers was considered. The data were stored in electronic spreadsheets using the EXCEL 2007 program and analyzed using the BioStat 5.0 and GraphPad Prism 9 program. In the univariate analysis, frequencies, measures of central tendency and dispersion were obtained, and for investigation of the hypotheses, the one-way ANOVA and Tukey tests were applied. All tests were performed considering a significance level of 5% (*p* < 0.05).

### 2.3. Ethical Aspects

The study was approved by the Research Ethics Committee of the Federal University of Pará, under the approval number 2.338.865.

## 3. Results

**Figure 1:**
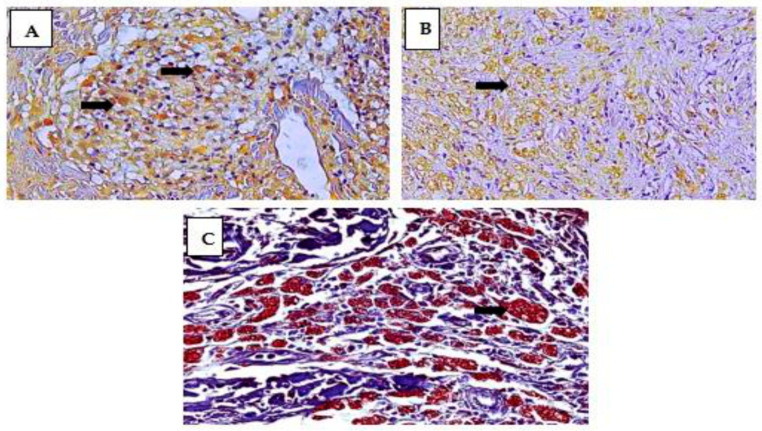
Immunostaining pattern of M1 (**A**), M2 (**B**), and M4 (**C**) macrophage subpopulations in leprosy lesion samples. (**A**) The M1 subpopulation characterized by the brownish labeling of iNOS in the cytoplasm of macrophages in the granuloma. (**B**) M2 macrophages expressing arginase-1 in the cytoplasm of these cells. (**C**) Double labeling of M4 macrophages for CD68 and MRP8, a hallmark of M4 macrophage subpopulations. Magnification: (**A**,**B**) 200× and (**C**) 400×.

The pattern of immunostaining for the macrophage subpopulations studied was characterized by the identification of brownish areas deposited in the cytoplasm or cell nucleus on the immunostained slides on a blue background stained with hematoxylin (Figure 1).

Quantitative analysis of the Mφ subpopulations (M1, M2, and M4) was performed based on three clinical forms of leprosy, namely VV, Borderline and TT. The numbers represent the average number of positive cells stained for each antibody, with the standard deviation. A statistically significant difference was observed between the three groups, as shown in Table 1.

The mean activity of MφM1 showed a statistically significant difference between VV (4.5 ± 1.3, *p* < 0.0001), Borderline (1.6 ± 0.4, *p* < 0.0001), and TT (12.5 ± 1.8, *p* < 0.0001) leprosy. Based on the MφM2 profile, a statistically significant difference was observed between VV (12.5 ± 2.3, *p* < 0.0001), Borderline (1.3 ± 0.2, *p* < 0.0001), and TT (3.2 ± 0.7, *p* < 0.0001) leprosy. Based on the MφM4 profile, a statistically significant difference was observed between VV (9.8 ± 1.7, *p* < 0.0001), Borderline (1.2 ± 0.2, *p* < 0.0001), and TT (2.6 ± 0.7, *p* < 0.0001) leprosy.

To improve characterization of Mφ levels in the three clinical forms, intra-lesion group as well as between-lesion comparisons were performed. In the intra-lesion group comparison, a greater number of cells were observed in the M2 profile in VV (*p* = 0.0001) and in the M1 profile in Borderline (*p* = 0.0001) and TT (*p* = 0.0001) leprosy.

In addition, for the comparison of Mφ levels between lesions, statistical significance was observed for MφM1 in the TT form (*p* = 0.0001) as well as MφM2 and MφM4 in the VV form (*p* = 0.0001 for both).

**Figure 2:**
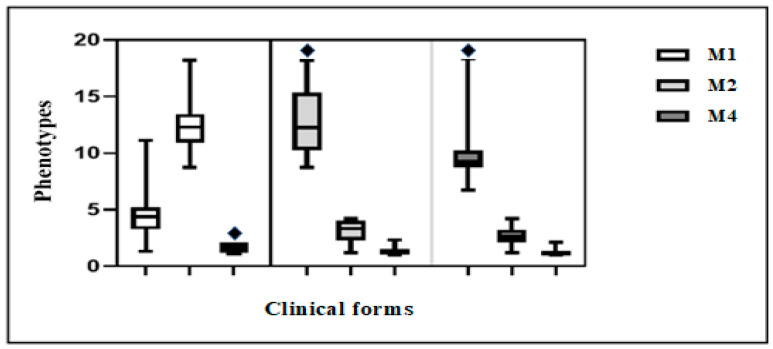
Boxplots of comparison between groups of M1, M2, and M4 macrophages. One-way analysis of variance (*p* < 0.05).

Intergroup comparison revealed a greater cell dispersion of MφM1 in the TT form (Figure 2). Among the groups, both MφM2 and MφM4 showed greater dispersion in the VV clinical form group.

**Figure 3:**
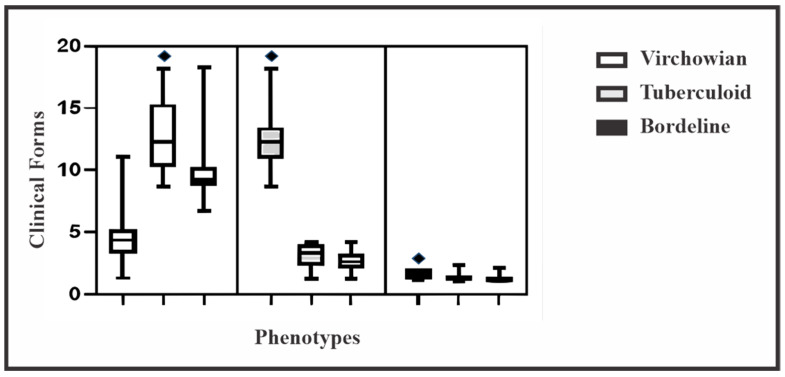
Boxplots for intragroup comparison of clinical forms VV, TT, and Borderline. One-way analysis of variance (*p* < 0.05).

Intragroup comparison revealed an enhanced dispersion of MφM2 and MφM4 in the VV group (Figure 3). Additionally, MφM1 were predominant in the TT group, whereas there was no evidence of Mφ polarization in Borderline.

The findings of the linear correlation between M1, M2 and M4 macrophage phenotypes and the three clinical forms of leprosy are presented in Table 2.

**Figure 4:**
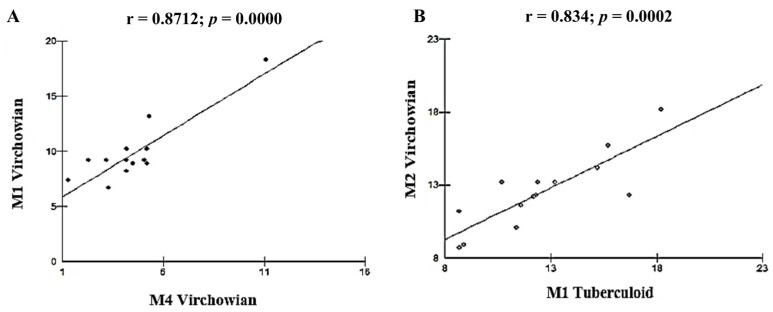
(**A**,**B**): Correlation analysis showing the strongest associations between the study variables.

Our findings indicated many strong correlations between the study variables. A strong, highly significant positive linear correlation was observed between VV M1 × VV M4 (r = 0.8712; *p* = 0.0000; Figure 4A) and between VV M2 × TT M1 (r = 0.834; *p* = 0.0002; Figure 4B).

**Figure 5:**
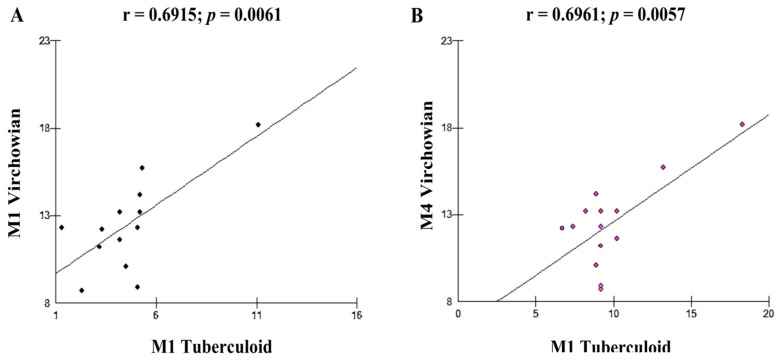
(**A**,**B**): correlation analysis showing the moderate association between VV and TT.

**Figure 6:**
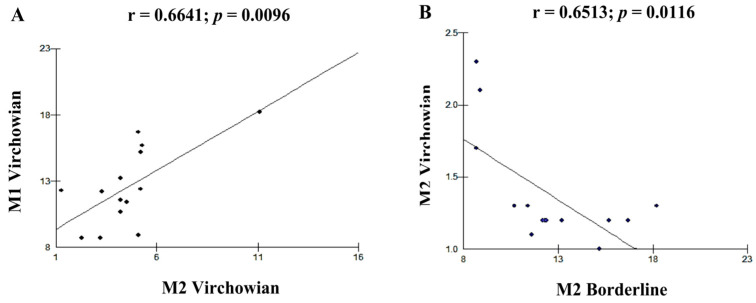
(**A**,**B**): correlation analysis showing moderate association between VV and Borderline.

**Figure 7:**
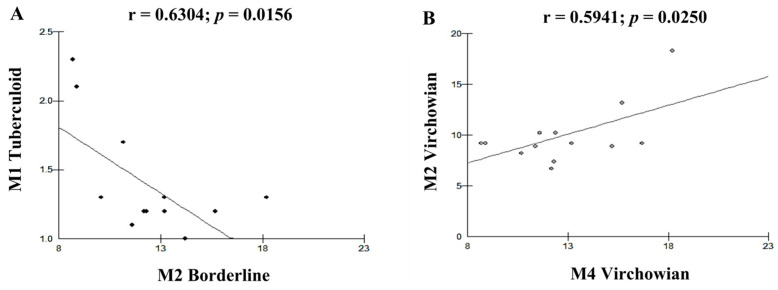
(**A**,**B**): correlation analysis showing the moderate associations among Borderline, TT, and VV.

Moderate linear correlations were observed for the VV M1 × M1 TT (r = 0.6915; *p* = 0.0061; Figure 5A), VV M4 × TT M1 (r = 0.6961; *p* = 0.0057; Figure 5B), VV M1 × VV M2 (r = 0.6641; *p* = 0.0096; Figure 6A), VV M2 × M2 Borderline (r = −0.6513; *p* = 0.0116; Figure 6B), TT M1 × M2 Borderline (r = 0.6304; *p* = 0.0156; Figure 7A), and VV M2 × VV M4 (r = 0.5941; *p* = 0.0250; Figure 7B) forms.

Other correlations between Mφ phenotypes and clinical forms of leprosy were moderate and weak (without statistical significance).

## 4. Discussion

Elucidation of the complex interaction between the tubercle bacillus and host remains a challenge for scientists, as it depends on the characteristics of virulence, evasion, and phenotype of the pathogen as well as factors related to the host’s immune response mechanisms [10,11,12,13].

Our findings revealed that patients diagnosed with TT were positive for the MφM1 phenotype (*p* < 0.0001) compared to those with other clinical forms of the disease.

MφM1 are known to possess high microbicidal potential and secrete pro-inflammatory cytokines 4. In the early stages of bacterial infection, Mφ are classically polarized to the M1 phenotype, which upregulates the expression of classic inflammatory phase markers, namely iNOS, IL-6, and TNF-α [10,14].

Elevated expression of stress response markers was observed in the M1 phenotype of TT lesions [15]. Real-time PCR assay has been employed to elucidate that the expression of CD38, Gpr18, and Fpr2 markers is exclusive to the M1 phenotype, and these markers are essential for cell activation and oxidative stress response mechanism [16,17].

During the pathogenesis of TT leprosy, IL-12 acts synergistically with IL-18 to increase the production of IFN-γ and ICAM3 via Th1 stimulation [18]. In leishmaniasis, which is also a spectral disease, IL-12 is essential for the development of an effective Th1 type of immune response [19]. The synergy between IL-1α and IL-12 serves to promote Th1 differentiation and prevent disease progression in BALB/c mice susceptible to *L. major* [20,21]. Sustained secretion of IL-12 is vital for the maintenance of the Th1 response associated with protection or disease progression in human leishmaniasis [22,23,24].

Immunohistochemical characterization of Mφs in the leprosy lesions remains an important pathological evidence of cell activation, oxidative stress response, Mφ polarization, and development of the pro-inflammatory response in leprosy [17,25]. Likewise, this evidence of tissue-protective response has already been demonstrated in a wide range of immunopathological studies on *M. leprae*, *H. pylori*, *M. tuberculosis*, *S. typhi*, and *C*. *trachomatis* [25,26,27].

Studies have shown that these pathogenic microorganisms employ several evasion mechanisms to suppress pro-inflammatory response and activate MφM2 in the host cells [26,28].

Our findings further showed that the M2 phenotype was significantly different in the VV clinical form of leprosy (*p* < 0.0001), indicating a strong anti-inflammatory response triggered by this subpopulation of Mφs. It was suggested that the high bacterial load in the cells not only enhances the chronicity of the VV form but also increases the presence of MφM2.

Previous studies on *H. pylori* revealed that patients with a higher bacterial load showed an increased population of MφM2 and that *H. pylori* survives within the megasomes [29,30].

In a study on chronic gastritis without preneoplastic lesions, the presence of MφM2 was correlated with bacterial density, and during the chronic phase, *H. pylori* infection persisted in the host [30]. This course of persistent *H. pylori* infection results in a large influx of Mφs, suggesting the inefficiency of the immune response [31,32,33]. The polarization of M1 and M2 macrophages was observed in the context of the pathogenesis of progressive renal interstitial fibrosis in mouses developed through the interaction of macrophages with TCD4 and TCD8 cells [34].

VV leprosy is characterized by an increase in anti-inflammatory cytokine levels, inhibition of Mφ activation, and the secretion of inflammatory mediators, which collectively enhance the survival of the bacillus via its immunosuppressive function [4,35,36]. Additionally, studies have shown that the increased level of kynurenine metabolites induces the differentiation of regulatory T cells (Treg) and the secretion of cytokines that enhance the anti-inflammatory profile of Mφs in the Virchowian pole [37,38].

Autophagic proteins are known to promote the formation of phagolysosomes and the consequent degradation of apoptotic cells, thereby releasing anti-inflammatory mediators and polarization of MφM2 [39]. During the symptomatic infectious phase of leprosy, an accumulation of pathogen-infected apoptotic cells occurs [40,41], eliciting an inflammatory microenvironment [40].

Studies have revealed strong evidence indicating that mannose-binding lectin (MBL) can facilitate the ingestion and spread of intracellular pathogens via opsonization [42,43]. Infection by *M. leprae* can lead to the development of the most widespread form of leprosy, known as lepromatous leprosy [44].

In leishmaniasis, oligosaccharides such as mannose and galactose are integrated into lipophosphoglycan structures [45], and play a significant role in the survival of parasites within phagolysosomes by participating in the oxidative responses of Mφs [46].

Our findings showed a positive correlation between the M2 phenotype of the VV form and the M1 phenotype of TT form (r = 0.834, *p* = 0.0001), indicating the behavior of IDO in the Virchowian pole, whereby the presence of CD68 in this pole could be associated with the migration of activated Mφs to the infected host cells [47].

This finding may provide insights into the various clinical aspects of neural damage and the management of leprosy via multidrug treatment strategy [48]. Clinical manifestation of leprosy reactions may occur in the TT and VV forms of the disease [49].

Mononeuritis multiplex is the most common presentation of TT leprosy [49]. Segmental demyelination is the predominant clinical evidence in Virchowian lesions, whereas Wallerian degeneration is frequently observed in tuberculoid lesions. Therefore, the complex interaction between bacilli and host immunological factors determines the activation of pro- or anti-inflammatory responses [49,50,51,52].

In the study on canine lymphomas, a greater number of macrophages was observed in high-grade and B-cell lymphomas; the latter also had the highest number of M1 and M2 macrophages. In those of high grade, macrophages are actively recruited and show a predominant M2 phenotype, which has been associated with protumor activity [53].

In the efferocytosis of apoptotic cells infected with *Streptococcus pneumoniae*, a mixed profile of simultaneously activated MφM1 and MφM2 was observed, whereas the efferocytosis of apoptotic cells infected with *Escherichia coli* induced the activation of the M1 phenotype [54].

Both necrosis and apoptosis have been shown to be important mechanisms of cell injury related to mycobacteria, including *M. leprae* and *M. tuberculosis* [11,55]. Pyroptosis may serve as additional cell death mechanism during the course of leprosy, responding to tissue injury, inhibiting Mφ differentiation, and inducing its death [56].

In the presence of mycobacteria, MφM1 undergo phenotypic changes to resemble MφM2, which exhibit increased expression of CD163 and SRA-I and enhanced phagocytic capacity. Therefore, in patients with paucibacillary leprosy, efferocytosis contributes to the persistence of the bacillus, increasing the population of MφM2 and sustaining the infection [57].

Several studies have characterized MφM2 as immune cells associated with anti-inflammatory response and tissue repair, whereas MφM4 are associated with activation of pro-inflammatory response, oxidative stress responses, and tissue repair [4,10].

Results of our study revealed a significant increase in the M4 subpopulation in VV leprosy (*p* < 0.0001). These data confirm the similarity between MφM4 that affect treatment response in leprosy and the immunosuppressive behavior of the host [10,58,59].

The phenotypic similarity of Mφs was validated in a study on Mφ phenotype modulation using CXCL4 (M4) and M-CSF (M2), whereby similar mRNA expression levels, leukocyte counts, and myeloid marker protein levels were observed. Transcriptome analysis was performed to confirm the strong correlation between phenotypes of Mφs (r = 0.934; *p* < 0.0001) [60].

Furthermore, we found an association between MφM1 and MφM4 (r = 0.871, *p* = 0.0001) in the VV form, indicating that in the absence of the CD163 receptor, the MφM4 exhibit a cytokine secretion profile similar to that of the MφM1. In a study on Mφ polarization associated with atheromatous intraplaque hemorrhages, hemoglobin-induced Mφ polarization led to increased expression of IL-10, high levels of the hemoglobin receptor inhibitor CD163, and low expression of HLA-DR [61].

Low CD163 expression has also been reported in other studies [60], and despite being the hallmark characteristic of M2 phenotype, it was observed that the chemokine CXCL4 induces an irreversible regulatory program of CD163 in MφM4 [62].

In the context of leprosy, this reduction in the costimulatory molecule levels enhances the formation of skin lesions [12,32]. This fact is evidenced in the clinical manifestations of the VV form, as the immune evasion of the bacillus enhances its proliferation and lesion formation [63,64].

Our findings reiterated that the M2 and M4 phenotypes are associated with the chronicity of the infection response and the ability of the immune cells to eliminate *M. leprae.* Additionally, these phenotypes could suppress oxidative stress-induced lipid degeneration, which is evidenced in the appearance of foamy Mφs with vacuoles filled with bacilli [65,66,67].

The increased expression of the M4 phenotype in unstable lesions suggests a strong correlation between the prevalence of MφM4 in human atherosclerotic plaques and their destabilization [68]. During a chronic condition such as atherosclerotic plaque, the M4 phenotype modulates the apoptosis of vascular smooth muscle cells, which contributes to plaque rupture [68]. These immune cells facilitate the formation of foamy Mφs via the accumulation of LDL, which is subsequently metabolized, causing harmful oxidative reactions, phagocytosis, and elimination of pathogens [62,69].

MMP7 is a well-defined marker of MφM4 [10]. MMPs can cleave growth factors to release active molecules [70], suppress immune responses after infection [71,72], and inactivate chemokine and inflammatory mediator secretion [73,74], thereby potentially affecting the host immune response to infection and cancer [75].

Our findings are in agreement with those of other studies, with regard to the functional aspects, phenotypic heterogeneity, and tissue immune response of Mφs in infectious diseases [10], as well as the M2 and M4 [11,12,13,76] phenotypes.

In this study, a strong correlation was observed between the clinical manifestations observed in the patients with the Mφ phenotypes for the pro- and anti-inflammatory phases. Therefore, we inferred that the phenotype of Mφs is determined by the surface markers present on them (MφM1: iNOS, IL-6, and TNF-α; MφM2: IL-10, CD163, and IL-13; and MφM4: CD68, MMP7, and MRP8).

The presence of specific biomarkers for Mφ activation in leprosy indicates the dynamic and transient nature of this immune cell type. Regulatory Mφs upregulate expression of several biologically important proteins, including MMP [75] and DC-STAMP, with the latter being expressed in stimulated Mφs [77] and implicated in cancer cell survival [78].

The increased expression of DC-STAMP following the stimulation via the presence of FcγR crosslinking is associated with increased phagocytosis and reduced antigen presentation and cytokine production [79,80], thereby promoting an anti-inflammatory environment similar to that in VV leprosy [75].

Simultaneous immunostaining of skin samples from patients diagnosed with the Borderline form of leprosy showed a predominant M1 phenotype (*p* < 0.0001) when compared to other clinical forms of the disease.

Analysis on NLRP1 and NLPR3 inflammasomes showed significant correlations between caspase-1 and IL-1β levels in Borderline leprosy, compared to the VV and TT forms of leprosy. The host–pathogen interaction determines the course of the adaptive immune response in leprosy [56].

Genes responsible for the formation and maintenance of granulomas and the activation and differentiation of helper T cells have been shown to bridge the gap between immune regulation and adaptive immunity. Small changes in these factors can alter the risk of developing leprosy or its severity [81].

Studies have identified genes that are differentially expressed after *M. leprae* stimulation, regardless of the Mφ polarization condition. Additionally, upon *M. leprae* stimulation, Mφ polarization upregulates the expression of numerous interferon-stimulated genes [82].

Activated MφM1 genes showed marked differential expression of genes involved in IFN type I regulation, Mφ activation, pathogen DNA recognition, and recruitment of effector cells to the inflammatory site in the presence of *M. leprae* genomic DNA [82]. Type I IFNs are associated with disseminated and progressive lepromatous lesions [83]. A recent study reported that different strains of *M. tuberculosis* elicit different NF-κB and IRF responses in human Mφs [84]. Therefore, further study on the modulation of Mφ phenotypic identity in the presence of diverse *M. leprae* strains is vital for knowledge on the immunopathogenesis of the disease.

## 5. Conclusions

Leprosy is considered a neglected disease that represents a serious public health problem in developing countries. In this study, three phenotypes of Mφs, namely M1, M2, and M4, were characterized based on their immunoexpression of iNOS, IL6, IL-10, IL-13, TGF-β, FGFb, TNF-α, CD68, CD63, arginase-1, MMP7, and MRP8. Our key findings on leprosy are summarized as follows.

First, MφM1 predominated in the clinical forms of Borderline (*p* = 0.0001) and TT (*p* = 0.0001). MφM1 played a pro-inflammatory role, evidenced by the immunoexpression of the TNF-α, IL-6, and iNOS markers. Based on the immunoexpression pattern of MφM1 in the three clinical forms of leprosy, our data showed predominance of MφM1 in the TT form (*p* < 0.0001). Statistically significant differences were observed between the means of the clinical forms VV, Borderline, and TT with the subpopulation of MφM1 (*p* < 0.0001).

Second, MφM2 were the predominant Mφs in the VV clinical form (*p* = 0.0001) and characterized by their immunoexpression pattern with the markers IL-10, IL-13, TGF-β, FGFb, CD163, and arginase-1. The immunoexpression pattern of the M2 phenotype was significantly different in the VV clinical form (*p* = 0.0001). There were statistically significant differences between the means of the clinical forms (VV, Borderline, and TT) and the subpopulation of MφM2 (*p* = 0.0001).

Third, MφM4 predominated the Mφ subpopulations in VV leprosy (*p* = 0.0001), indicating the association between MφM4 and chronic pathological processes in leprosy. The pro-inflammatory function of MφM4 was indicated in their positive immunoexpression pattern for TNF-α, IL-6, CD68, MMP7, and MRP8. The immunoexpression pattern of the M4 phenotype was significantly correlated to the VV form of leprosy (*p* = 0.0001). ANOVA revealed statistically significant differences between the means of the three clinical forms (VV, Borderline and TT) and the subpopulation of MφM4 (*p* = 0.0001).

Fourth, our data also revealed a highly significant positive linear correlation between VV M1 × VV M4 (r = 0.8712; *p* = 0.0000), as well as between VV M2 × TT M1 (r = 0.834; *p* = 0.0002).

Our findings revealed the relationship of macrophage subpopulations and their profile according to the respective clinical forms in persistent pathological processes of the inflammatory response during the disease. This makes it possible for new studies to advance in the diagnosis and personalized treatment of leprosy.

## Figures and Tables

**Table 1:** Quantitative analysis of macrophage profiles according to the clinical form of leprosy.

Macrophage Phenotype	Clinical Forms			*p* Value *
	Virchowian Mean ± SD (95% CI)	Borderline Mean ± SD (95% CI)	Tuberculoid Mean ± SD (95% CI)	
M1	4.5 ± 1.3 (0.7–5.2)	1.6 ± 0.4 (0.2–1.8)	12.5 ± 1.8 (0.9–13.4)	<0.0001
M2	12.5 ± 2.3 (1.2–13.7)	1.3 ± 0.2 (0.1–1.5)	3.2 ± 0.7 (0.3–3.5)	<0.0001
M4	9.8 ± 1.7 (0.9–10.7)	1.2 ± 0.2 (0.1–1.3)	2.6 ± 0.7 (0.3–3.0)	<0.0001
*p* value*	<0.0001	<0.0001	<0.0001	

Test: ANOVA. SD: standard deviation, 95% CI: 95% confidence interval. * One-way analysis of variance (*p* < 0.05).

**Table 2:** Linear correlation between M1, M2, and M4 macrophage phenotypes and the three clinical forms of leprosy.

Cell Profile	r (Pearson)	*p* (Value)
M1V–M4V	0.8712	<0.0001 ^A^
M2V–M1T	0.8341	0.0002 ^A^
M1V–M1T	0.6915	0.0061 ^B^
M4V–M1T	0.6961	0.0057 ^B^
M1V–M2V	0.6641	0.0096 ^B^
M2V–M2I	−0.6513	0.0116 ^B^
M1T–M2I	−0.6304	0.0156 ^B^
M2V–M4V	0.5941	0.0250 ^B^
M2T–M2I	−0.4505	0.1059
M4T–M2I	−0.4471	0.1089
M2I–M4I	0.4299	0.1249
M1T–M4T	0.4111	0.1442
M2V–M4T	0.4014	0.1548
M2V–M2T	0.3398	0.2345
M2V–M4I	−0.3367	0.2391
M1T–M2T	0.3249	0.2570
M1V–M1I	0.313	0.2758
M2T–M4T	0.2956	0.3048
M1T–M4I	−0.2866	0.3204
M1I–M4I	0.2695	0.3514
M1V–M2T	0.2679	0.3544
M2T–M1I	0.2383	0.4120
M2T–M4I	−0.2104	0.4703
M4T–M4I	−0.2095	0.4723
M1V–M2I	−0.2011	0.4906
M1V–M4T	0.188	0.5198
M4V–M2T	0.187	0.5221
M4V–M1I	0.1835	0.5301
M4V–M4I	−0.1249	0.6704
M2V–M1I	−0.1006	0.7322
M4V–M4T	0.0949	0.7470
M1I–M2I	0.0939	0.7495
M4V–M2I	−0.0637	0.8286
M1V–M4I	0.046	0.8758
M4T–M1I	−0.034	0.9081
M1T–M1I	−0.0193	0.9477

**^A^** Strong linear correlations. **^B^** Moderate linear correlations.

## Data Availability

The database used and/or analyzed during the current study is not publicly accessible but can be available, upon reasonable request, from the corresponding authors.
